# Foot-and-Mouth Disease Virus Structural Protein VP1 Destroys the Stability of the TPL2 Trimer by Degradation of TPL2 To Evade Host Antiviral Immunity

**DOI:** 10.1128/JVI.02149-20

**Published:** 2021-03-10

**Authors:** Keshan Zhang, Minghao Yan, Junhong Hao, Chaochao Shen, Zixiang Zhu, Dajun Zhang, Jing Hou, Guowei Xu, Dan Li, Haixue Zheng, Xiangtao Liu

**Affiliations:** a State Key Laboratory of Veterinary Etiological Biology, National Foot-and-Mouth Disease Reference Laboratory, Lanzhou Veterinary Research Institute, Chinese Academy of Agriculture Science, Lanzhou, China; Instituto de Biotecnologia/UNAM

**Keywords:** FMDV, TPL2, VP1, trimer complex, immune escape, foot-and-mouth disease virus

## Abstract

Tumor progression locus 2 (TPL2) is a serine/threonine kinase that belongs to the mitogen-activated protein 3 kinase (MAP3K) family, and it plays an important role in pathogen infection. The trimer complex of TPL2, p105, and ABIN2 is essential for maintenance of TPL2 steady-state levels and host cell response to pathogens. Foot-and-mouth disease virus (FMDV) is a positive-strand RNA virus of the family *Picornaviridae* that encodes proteins capable of antagonizing host immune responses to achieve infection. The VP1 protein of FMDV is a multifunctional protein that can bind host cells and induce an immune response as well as cell apoptosis. However, the role and mechanisms of TPL2 in FMDV infection remain unknown. Here, we determined that FMDV infection could inhibit TPL2, p105, and ABIN2 at the transcription and protein levels, while VP1 could only inhibit TPL2, p105, and ABIN2 at the protein level. TPL2 inhibited the replication of FMDV *in vivo* and *in vitro*, and the 268- to 283-amino-acid region in the TPL2 kinase domain was essential for interaction with VP1. Moreover, VP1 promoted K48-linked polyubiquitination of TPL2 and degraded TPL2 by the proteasome pathway. However, VP1-induced degradation of p105 and ABIN2 was independent of proteasome, autophagy, lysosome, and caspase-dependent pathways. Further studies showed that VP1 destroyed the stability of the TPL2-p105-ABIN2 complex. Taken together, these results revealed that VP1 antagonized TPL2-meditated antivirus activity by degrading TPL2 and destroying its complex. These findings may contribute to understanding FMDV-host interactions and improving the development of a novel vaccine to prevent FMDV infection.

**IMPORTANCE** Virus-host interactions are critical for virus infection. This study was the first to demonstrate the antiviral effect of host TPL2 during FMDV replication by increasing production of interferons and antiviral cytokines. Both FMDV and VP1 protein can reduce host TPL2, ABIN2, and p105 to destroy the TPL2-p105-ABIN2 trimer complex. VP1 interacted with TPL2 and degraded TPL2 via the proteasome pathway to repress TPL2-mediated antivirus activity. This study provided new insights into FMDV immune evasion mechanisms, elucidating new information regarding FMDV counteraction of host antivirus activity.

## Abstract (precis)

Virus-host interactions are critical for virus infection. This study was the first to demonstrate the antiviral effect of host TPL2 during FMDV replication by increasing production of interferons and antiviral cytokines.

## INTRODUCTION

Foot-and-mouth disease is a severe infectious disease of cloven-hoofed animals caused by foot-and-mouth disease virus (FMDV) infection, which seriously harms the development of animal husbandry (1–3). FMDV is a single-stranded positive-strand RNA virus belonging to the *Aphthovirus* genus of the *Picornaviridae* family. Seven serotypes of FMDV (O, A, C, SAT1, SAT2, SAT3, and Asia1) and multiple subtypes are currently recognized (4, 5). The FMDV genome is approximately 8.5 kb in length and consists of a 5′ untranslated region (UTR), a complete open reading frame (ORF), and a 3′UTR with a poly(A) tail (6). The ORF-encoded polyprotein is cleaved into four structural proteins (VP1, VP2, VP3, and VP4) and eight nonstructural proteins (L, 2A, 2B, 2C, 3A, 3B, 3C, and 3D) by viral self-encoded proteases (7, 8). These viral proteins perform various roles in the pathogenicity of FMDV and can block the functions of various host proteins to counteract host antiviral responses.

Tumor progression locus 2 (TPL2) is a serine/threonine kinase that regulates the production of host interferons and cytokines (9), and it is an important participant in the process of inflammation and cancer (10, 11). TPL2 consists of an amino acid terminal region of unknown function, a serine/threonine kinase domain, and a carboxyl-terminal region with a determinant “degron” sequence (12, 13). Under normal circumstances, TPL2 forms a trimer complex with p105 and A20-binding inhibitor of NF-κB activation 2 (ABIN2) in a stable state, where TPL2 cannot perform its biological function (14, 15). The downstream inhibitor of κB (IκB) kinase (IKK) complex is activated when tumor necrosis factor receptor (TNFR), toll-like receptors (TLR), and interleukin-1 receptor (IL-1R) are stimulated, leading to the degradation of p105 and releasing TPL2 (9, 16–19). TPL2 is then phosphorylated and activated. Activated TPL2 activates nodal proteins via downstream signaling pathways to perform biological functions (20–23). Recent studies have shown that TPL2 is also an important host immunoregulatory protein involved in innate and adaptive immunity, and it can resist the invasion of foreign pathogens. However, the role of TPL2 during FMDV infection remains unknown.

During the process of evolution, FMDV has accumulated a variety of self-encoded proteins to antagonize the host innate immune response. The virus effectively forms a unique immune escape mechanism that enables it to survive the host antiviral defense mechanism. For example, FMDV 2B negatively regulates retinoic acid-inducible gene I (RIG-I)-like receptor (RLR)-mediated induction of beta interferon (IFN-β) by inhibiting phosphorylation of TANK-binding kinase 1 (TBK1) and interferon regulatory factor 3 (IRF3) (24); FMDV 3C protease induces the degradation of host protein kinase R (PKR) through the lysosomal pathway, thereby promoting viral replication (25); and FMDV L significantly inhibits the ubiquitination of RIG-I, TBK1, TNFR-associated factor 6 (TRAF6), and TRAF3, thereby inhibiting the induction of type I IFN (26). FMDV structural protein VP1 has the function of binding to host cell receptors, and it contributes to the host-cell interaction through identified cavities on its surface (27) that are the key to virus infection. The interaction between VP1 and host proteins also largely determines the course of the disease. Our previous studies have determined that VP1 and host TPL2 interact (28), but the regulatory effects of VP1 on TPL2 and its mechanism are still unknown.

The present study verified the role of TPL2 in the process of FMDV infection *in vivo* and *in vitro* and determined its antiviral effect on FMDV. TPL2 inhibited FMDV replication by promoting the production of FMDV-induced IFN and other antiviral cytokines. Further studies demonstrated that FMDV induced a decrease in the expression of host TPL2 in a manner dependent on VP1. The VP1 protein specifically interacted with TPL2, promoted K48-linked polyubiquitination of TPL2, and then degraded TPL2 through the proteasome pathway. This affected the stability of the trimer and resulted in a decrease of the amount of TPL2 released from the trimer. This precluded TPL2 downstream signaling pathways and subsequent production of antiviral cytokines, thereby increasing FMDV replication. Thus, we describe a new mechanism for FMDV to antagonize host innate immune response through a virus-encoded protein and deepen our understanding of host innate immunity and the pathogenic mechanism of FMDV.

## RESULTS

### TPL2 inhibits FMDV replication during virus infection.

**FIG 1:**
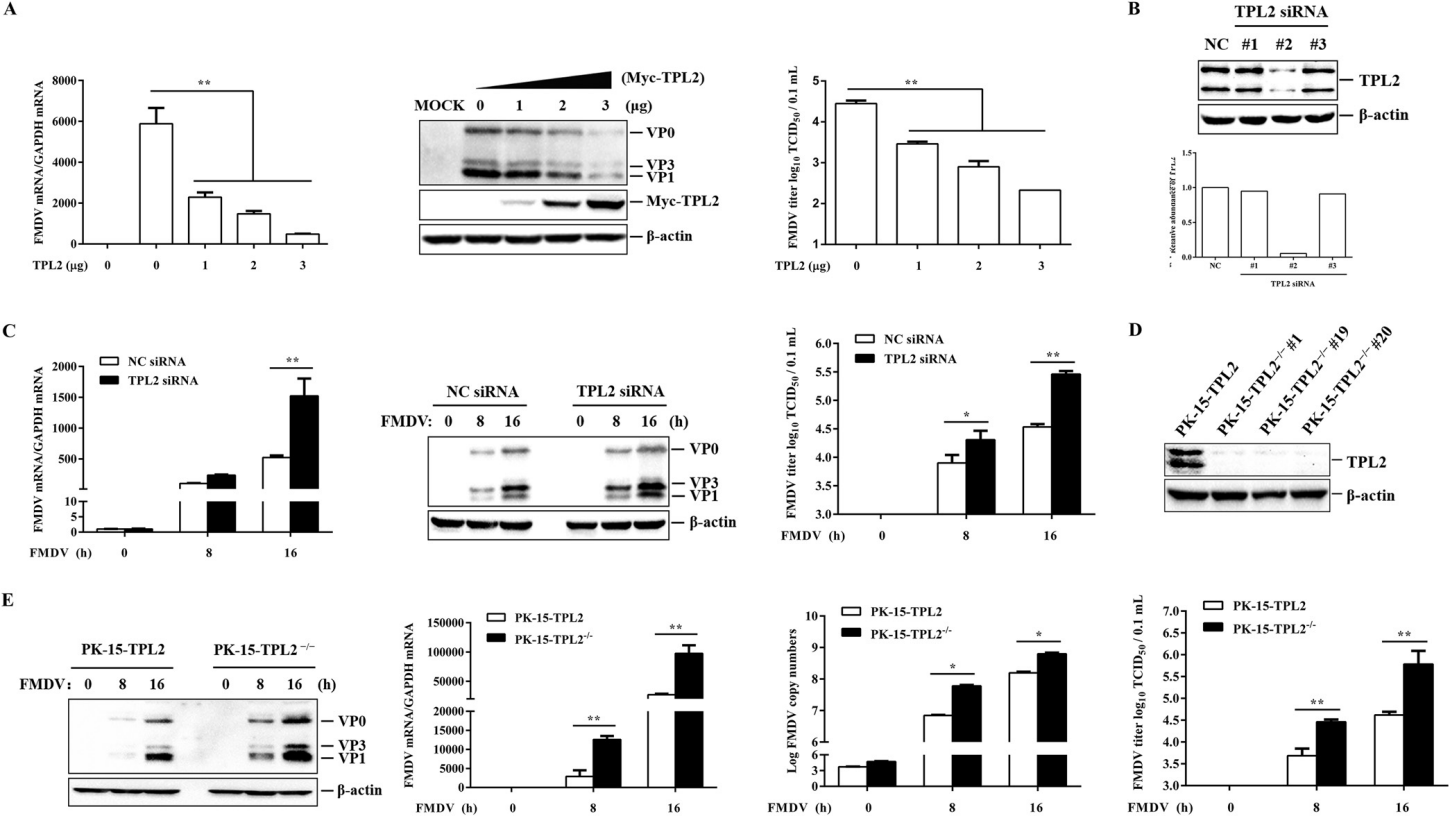
TPL2 played an antiviral role during FMDV replication. (A) PK-15 cells were transfected with increasing amounts of Myc-tagged TPL2-expressing plasmid (0, 1, 2, or 3 μg), and the empty Myc vector (3, 2, 1, or 0 μg) was used in the transfection process to ensure that the cells received the same amounts of total DNA plasmids. At 24 hpt, the cells were infected with equal amounts of FMDV (MOI of 1) for 12 h. The replication levels of FMDV were measured by RT-qPCR, and the expression levels of Myc-TPL2 and viral proteins were detected by Western blotting. A rabbit anti-FMDV polyclonal antibody was used to confirm the expression of viral proteins. Western blotting showed three protein bands of VP0 (40 kDa), VP1 (25 kDa), and VP3 (26 kDa). The viral titers were measured by 50% tissue culture infective dose (TCID_50_) assay. (B) PK-15 cells were transfected with 100 pM NC siRNA or TPL2 siRNA for 48 h. The knockdown efficiency was determined by Western blotting. Relative fold change in abundance of TPL2 protein was determined by densitometric analysis. (C) PK-15 cells were transfected with 100 pM NC siRNA or TPL2 siRNA for 48 h, followed by infection with equal amounts of FMDV (MOI of 1) for 0, 8, and 16 h. The replication levels of FMDV were measured by RT-qPCR, and the expression levels of endogenous TPL2 and viral proteins were detected by Western blotting with anti-TPL2 antibody and anti-FMDV antibody. The viral titers were measured by 50% tissue culture infective dose (TCID_50_) assay. (D) PK-15-TPL2 or PK-15-TPL2^−/−^ cells were seeded in six-well culture plates for 24 h, and TPL2 protein was detected by Western blotting. (E) PK-15-TPL2 and PK-15-TPL2^−/−^ cells were infected with equal amounts of FMDV (MOI of 1) for 0, 8, and 16 h. The expression levels of viral proteins were detected by Western blotting. The replication levels of FMDV and viral copy numbers were measured by RT-qPCR, and the viral titers were measured by TCID_50_ assay.

The role of TPL2 in FMDV infection has remained unknown. To determine whether TPL2 is involved in FMDV replication, gain-of-function and loss-of-function assays were performed. PK-15 cells were transfected with increased doses of Myc-TPL2 plasmids, and the empty vector was used in the transfection process to ensure that the cells received the same amounts of total plasmids. At 24 h posttransfection (hpt), the cells were infected with FMDV at a multiplicity of infection (MOI) of 1 for 12 h. Viral RNA levels, viral protein abundance, and viral titers were then compared. Quantitative PCR (qPCR), Western blotting, and viral titer analysis showed that overexpression of TPL2 significantly suppressed FMDV replication in a dose-dependent manner (Fig. 1A).

The replication status of FMDV in TPL2-downregulated cells was also evaluated. Three TPL2 short interfering RNAs (siRNAs) were designed and constructed, and their silencing efficiencies were evaluated by Western blotting in PK-15 cells. The #2 TPL2 siRNA, with the best inhibition of TPL2 expression, was selected for subsequent experiments (Fig. 1B). Thus, TPL2 was knocked down in PK-15 cells by transfection of TPL2 siRNA. Viral RNA levels, viral protein abundance, and viral titers in the TPL2 siRNA cells were analyzed and compared with the negative-control (NC) siRNA cells at 0, 8, or 16 h postinfection (hpi). The results demonstrated that FMDV replication was significantly enhanced in TPL2 siRNA cells compared with that of NC siRNA-treated cells (Fig. 1C).

To further evaluate the relationship between TPL2 and FMDV replication, TPL2 knockout PK-15 cells (PK-15-TPL2^−/−^) were established by using the CRISPR/Cas9 system. Western blotting demonstrated the successful knockout of TPL2 in the established cell line (Fig. 1D). The control cell line (PK-15-TPL2) was also obtained in parallel. PK-15-TPL2^−/−^ and PK-15-TPL2 cells were infected with equal amounts of FMDV (MOI of 1). Viral copy numbers, viral RNA levels, viral protein abundance, and viral titers were determined and compared at the indicated time points after virus infection. As shown in Fig. 1E, the expression of viral protein and RNA as well as viral copy numbers and viral titers were significantly higher in the PK-15-TPL2^−/−^ cells than in the PK-15-TPL2 cells. Taken together, these results conclusively demonstrated the important antiviral role for TPL2 in FMDV infection.

### TPL2 positively regulates FMDV-triggered induction of downstream antiviral cytokines.

**FIG 2:**
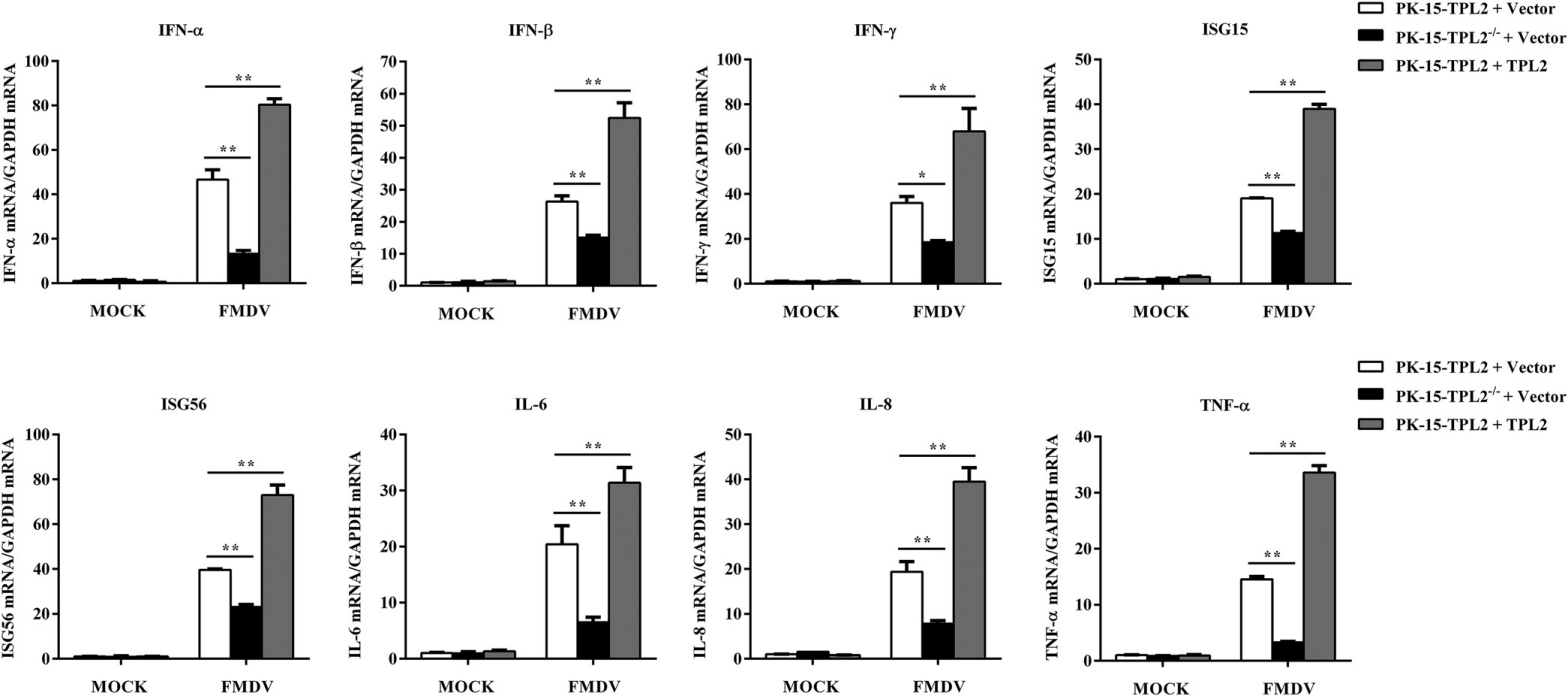
TPL2 enhanced antiviral effectors induced by FMDV infection in PK-15 cells. PK-15-TPL2 and PK-15-TPL2^−/−^ cells were transfected with Myc-TPL2-expressing plasmid (3 μg) or empty Myc vector (3 μg) for 24 h and then mock infected or infected with equal amounts of FMDV (MOI of 1) for 12 h. The mRNA expression levels of IFN-α, IFN-β, IFN-γ, ISG15, ISG56, IL-6, IL-8, and TNF-α were measured by RT-qPCR assay.

To reveal how TPL2 exerts antiviral effects during FMDV infection, we investigated the effect of TPL2 on the expression of IFN, IFN-stimulated genes (ISG), and other downstream antiviral genes during FMDV infection. PK-15-TPL2 and PK-15-TPL2^−/−^ cells were transfected with Myc-TPL2 or empty Myc vector for 24 h. The cells were then infected with equal amounts of FMDV (MOI of 1) for 12 h. Reverse transcription-qPCR (RT-qPCR) analysis indicated that the transcription of IFN-α, IFN-β, IFN-γ, ISG15, ISG56, IL-6, IL-8, and TNF-α genes were significantly decreased in PK-15-TPL2^−/−^ cells compared with those of the PK-15-TPL2 cells after FMDV infection (Fig. 2). Conversely, overexpression of TPL2 significantly increased the mRNA levels of downstream genes, including IFN-α, IFN-β, IFN-γ, ISG15, ISG56, IL-6, IL-8, and TNF-α, induced by FMDV infection (Fig. 2). These results indicated that TPL2 positively regulated FMDV-triggered induction of antiviral cytokines.

### TPL2 is essential for innate immune responses to FMDV *in vivo*.

**FIG 3:**
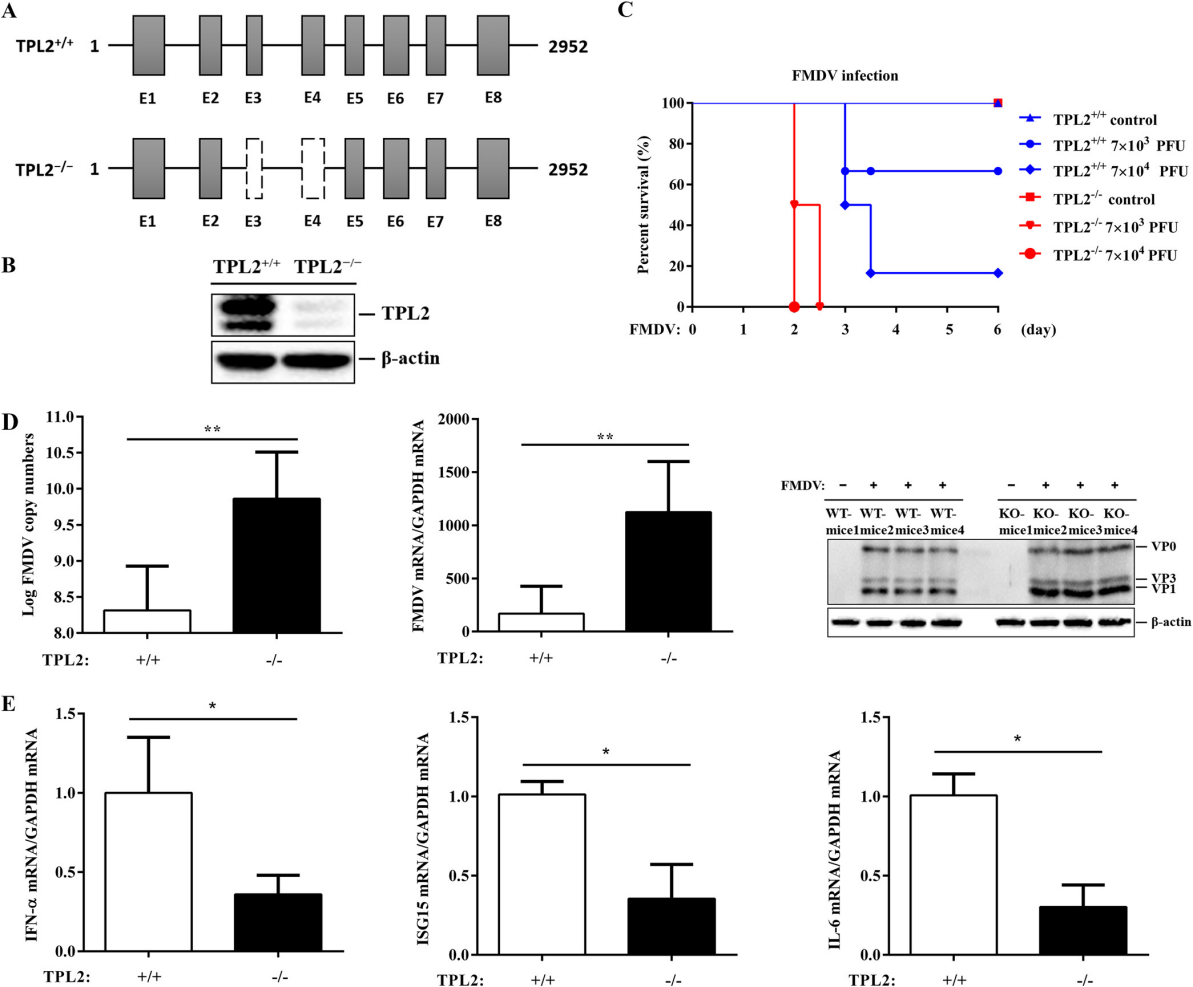
TPL2 was essential for host against FMDV infection in mice. (A) Knockout (KO) schematic representation position of the sequence for TPL2 in TPL2^−/−^ mice. The mouse genome of TPL2 contained eight exons. CRISPR/Cas9 technology was used to design a targeted vector to delete exon 3 and exon 4. (B) Protein levels of TPL2 in TPL2^+/+^ and TPL2^−/−^ mice were detected by Western blotting to determine TPL2 knockout efficiency. (C) Three-day-old TPL2^+/+^ (*n* = 6 in each group) and TPL2^−/−^ (*n* = 4 in each group) suckling mice were used and divided into three groups. One group was subcutaneously injected with equal amounts of PBS, and the other two groups were injected subcutaneously with two concentrations of FMDV (7 × 10^3^ and 7 × 10^4^ PFU). The survival rate was monitored every 12 h. (D) Three-day-old TPL2^+/+^ (*n* = 4 in each group) and TPL2^−/−^ (*n* = 4 in each group) mice were used and divided into two groups in total. Two groups were subcutaneously injected with equal amounts of PBS and FMDV (7 × 10^4^ PFU) and euthanized at 72 h after FMDV infection. Samples from FMDV-infected mouse were detected by RT-qPCR individually. Samples from mock-infected mice were mixed, and samples from infected mice were left unmixed and then analyzed by Western blotting. The replication levels of FMDV were measured by RT-qPCR. The expression levels of FMDV proteins in mice were detected by Western blotting with rabbit anti-FMDV polyclonal antibody. Each lane represents different FMDV-infected suckling mice. +/+, wild-type suckling mice; −/−, TPL2 knockout suckling mice. (E) The mRNA expression levels of IFN-α, ISG15, and IL-6 in mice were measured by RT-qPCR for the samples from panel D. The variance represents the difference between different mice.

To further unveil the functions of TPL2 in FMDV-triggered innate immune response, we deleted 3 and 4 exons in mice using CRISPR/Cas9 systems, generating TPL2 knockout mice (Fig. 3A). Western blotting demonstrated that the expression of TPL2 protein was effectively deleted in TPL2^−/−^ mice (Fig. 3B). These TPL2^−/−^ mice were born at a standard Mendelian ratio and did not show any developmental or behavioral abnormality compared with their wild-type (WT) littermates. We infected 3-day-old TPL2^+/+^ and TPL2^−/−^ suckling mice with FMDV by subcutaneous injection and monitored their survival. The results indicated that TPL2^−/−^ suckling mice were more susceptible to FMDV-induced death than their WT littermates (Fig. 3C). It is calculated that the 50% lethal dose (LD_50_) of TPL2 knockout suckling mice and wild-type suckling mice were 7.625 and 6.498, respectively; levels for TPL2 knockout suckling mice are about 14 times higher than those for the wild-type suckling mice (data not shown). Consistent with this, the ketone bodies of lactated mice were taken 48 h after infection to measure the replication of FMDV in suckling mice, and the TPL2^−/−^ suckling mice infected with FMDV showed higher FMDV viral copy numbers, RNA levels, and protein abundance than infected WT littermates (Fig. 3D). In addition, the levels of internal antiviral genes, including IFN-α, IL-6, and ISG15, were much lower in TPL2^−/−^ suckling mice than in their WT littermates after FMDV infection (Fig. 3E). These results indicated that TPL2 was essential for host defense against FMDV infection *in vivo*.

### FMDV infection inhibits TPL2 expression.

**FIG 4:**
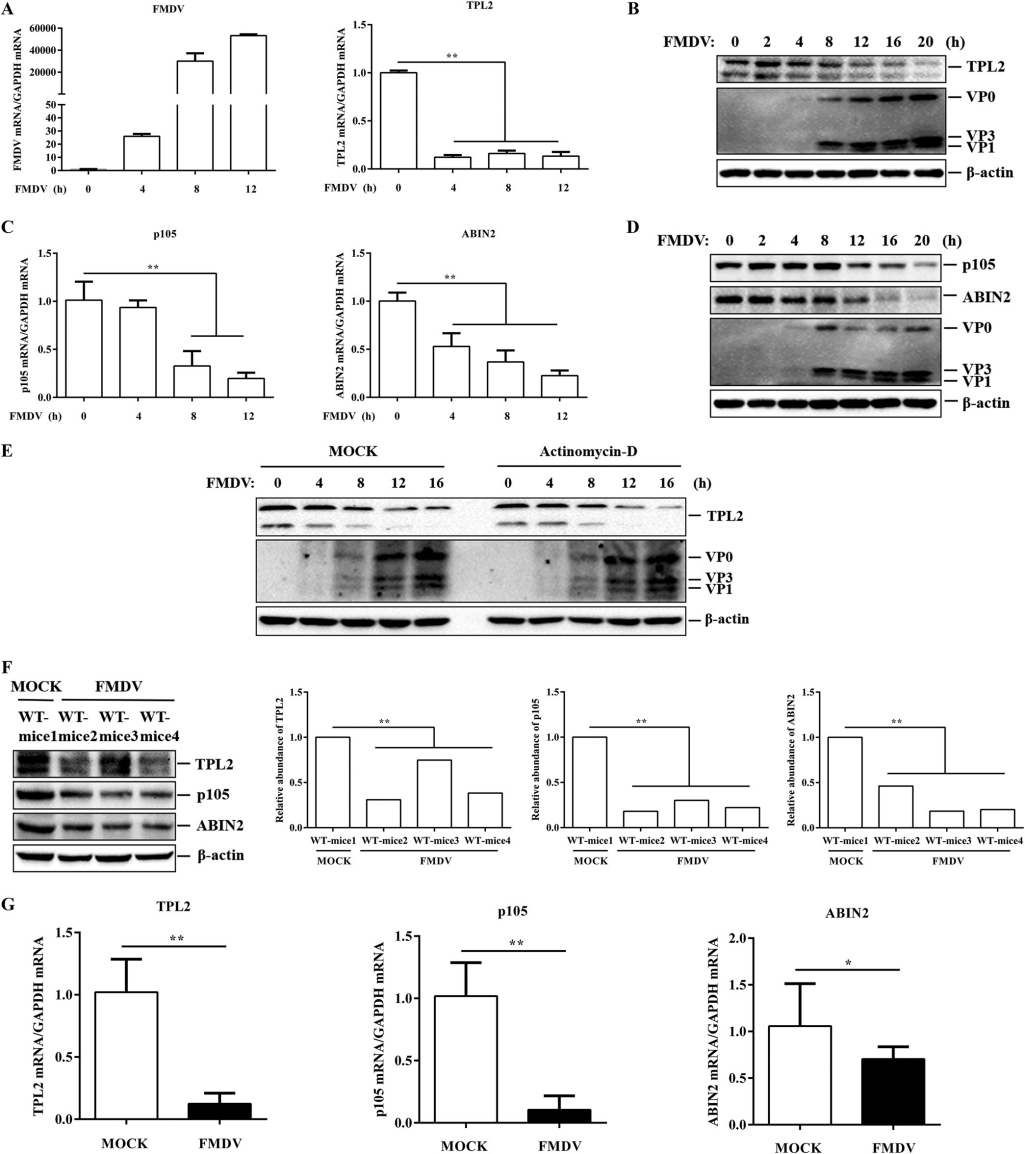
TPL2, ABIN2, and P105 levels were decreased after FMDV infection *in vivo* and *in vitro*. (A) PK-15 cells were infected with equal amounts of FMDV (MOI of 1) for 0, 4, 8, or 12 h. The transcriptional levels of FMDV and TPL2 were measured by RT-qPCR. (B) PK-15 cells were infected with equal amounts of FMDV (MOI of 1) for 0, 2, 4, 8, 12, 16, and 20 h. The expression levels of endogenous TPL2 and viral proteins were detected by Western blotting with anti-TPL2 and anti-FMDV antibodies. (C) The mRNA expression levels of p105 and ABIN2 were measured by RT-qPCR for the samples from panel A. (D) The expression levels of endogenous p105, ABIN2, and viral proteins were detected by Western blotting for the samples from panel B with anti-p105, anti-ABIN2, and anti-FMDV antibodies. (E) PK-15 cells with or without actinomycin D (10 μg/ml) treatment were infected with equal amounts of FMDV (MOI of 1) for 0, 4, 8, 12, and 16 h. The expression levels of endogenous TPL2 and viral proteins were detected by Western blotting. (F and G) Three-day-old wild-type mice (*n* = 4 in each group) were divided into two groups. Two groups were subcutaneously injected with equal amounts of FMDV (7 × 10^4^ PFU) and PBS and then euthanized 72 h after FMDV infection. All Western blotting samples from suckling mice without FMDV infection were mixed, and all Western blotting samples of suckling mice infected with FMDV were not mixed. (F) Expression levels of TPL2, p105, and ABIN2 proteins in mice were detected by Western blotting with anti-TPL2, anti-p105, and anti-ABIN2 antibodies. Relative fold change in abundance of TPL2, p105, and ABIN2 protein was determined by densitometric analysis. WT-mice1, all combinations of noninfected FMDV suckling mice; WT-mice2, WT-mice3, and WT-mice4, three suckling mice infected with FMDV. (G) RT-qPCR samples of uninfected suckling mice and FMDV-infected suckling mice were not mixed. The transcriptional levels of TPL2, p105, and ABIN2 in mice were measured by RT-qPCR.

Because TPL2 plays an antiviral role in FMDV infection, it is necessary to investigate the state of TPL2 during FMDV infection. PK-15 cells were infected with equal amounts of FMDV (MOI of 1), and the dynamics of TPL2 were determined over time. The results showed that TPL2 transcription levels were significantly downregulated as the infection progressed (Fig. 4A), while viral RNA was gradually increased, confirming the correlation between TPL2 expression and viral replication (Fig. 4A). We also detected the abundance of TPL2 protein in FMDV-infected cells and found that the protein levels of TPL2 gradually decreased as infection progressed (Fig. 4B). To investigate whether FMDV infection reduced the expression of p105 and ABIN2, two other proteins that form a trimer complex with TPL2, we infected PK-15 cells as described above and determined the state of the two proteins after FMDV infection. As shown in Fig. 4C and D, the transcription and protein levels of p105 and ABIN2 decreased significantly as infection progresses. It was observed that the transcription and translation methods of TPL2 were different after FMDV infection. To further confirm the inhibitory activity of FMDV on TPL2 expression, the PK-15 cells were maintained in the culture medium in the presence or absence of actinomycin D and then infected with equal amounts of FMDV (MOI of 1), and the expression level of TPL2 protein was measured over time. Actinomycin D is a specific inhibitor of cellular transcription. As shown in Fig. 4E, the protein levels of TPL2 showed a more linear decrease with time in both actinomycin D-treated and untreated cells. This implied that FMDV has different inhibitory mechanisms on TPL2 transcription and protein levels.

To further verify the above-described results, we conducted *in vivo* experiments in suckling mice. The suckling mice were infected with FMDV by subcutaneous injection, and the transcription and protein expression levels of TPL2, p105, and ABIN2 were detected. The results demonstrated that FMDV infection reduced the expression of TPL2, p105, and ABIN2 proteins (Fig. 4F). In addition, the transcription levels of TPL2, p105, and ABIN2 were significantly downregulated after FMDV infection (Fig. 4G). Taken together, these results indicated that FMDV infection inhibited the expression of TPL2, p105, and ABIN2.

### FMDV VP1 protein inhibits TPL2 protein expression.

**FIG 5:**
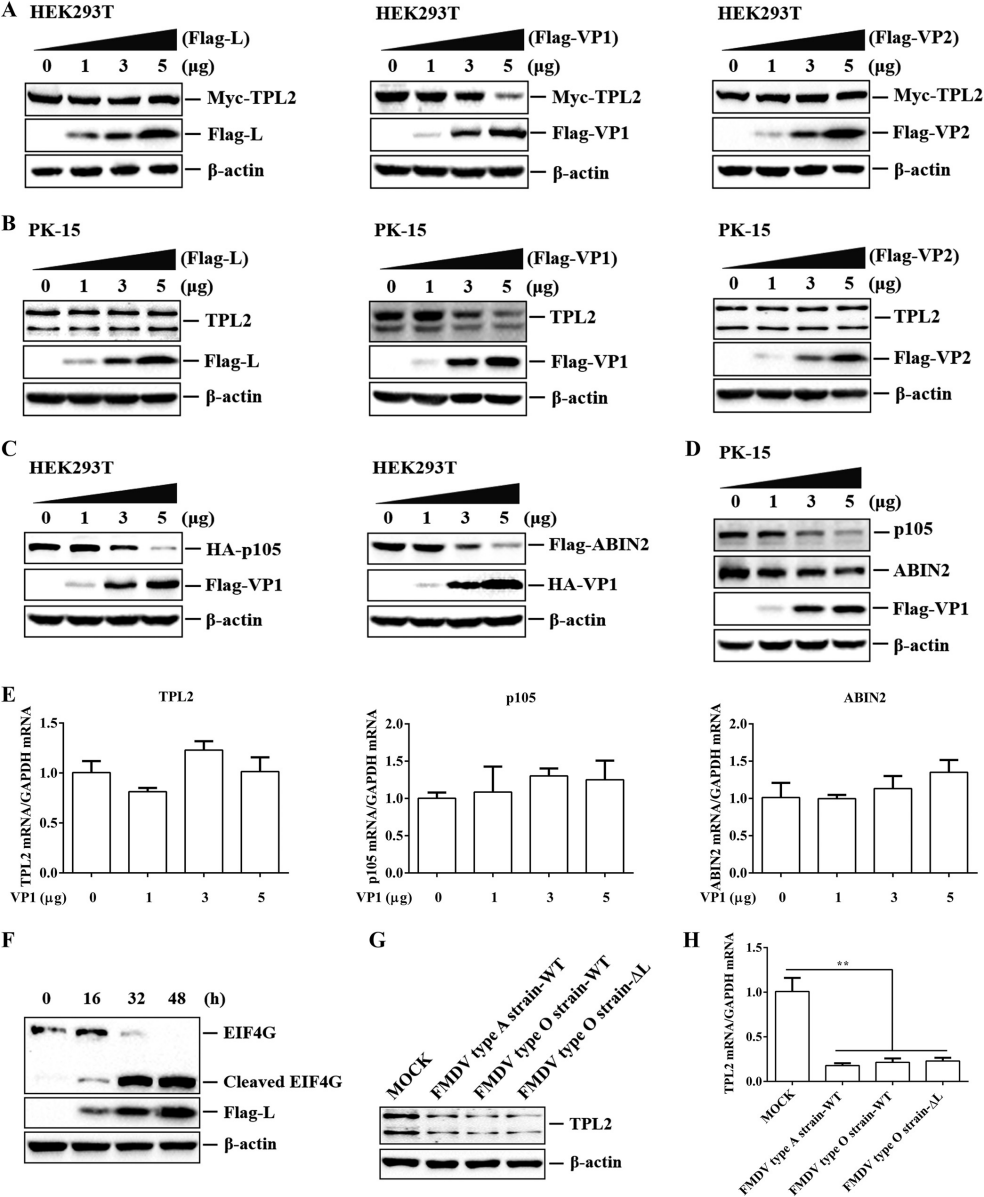
FMDV VP1 protein was responsible for reducing the expression of TPL2, p105, and ABIN2 at protein level but not transcription level. (A) HEK293T cells were cotransfected with Myc-TPL2 (1 μg), along with increasing amounts of Flag-L-, Flag-VP1-, or Flag-VP2-expressing plasmid (0, 1, 3, and 5 μg), and the empty Flag vector (5, 4, 2, and 0 μg) was used in the transfection process. At 24 hpt, the expression levels of Myc-TPL2 and Flag-tagged viral proteins (L, VP1, and VP2) were analyzed by Western blotting with anti-Myc and anti-Flag antibodies. (B) PK-15 cells were transfected with increasing amounts of Flag-L-, Flag-VP1-, or Flag-VP2-expressing plasmid (0, 1, 3, and 5 μg), and the empty Flag vector (5, 4, 2, and 0 μg) was used in the transfection process. At 24 hpt, the expression levels of endogenous TPL2 and Flag-tagged viral proteins were analyzed by Western blotting with anti-TPL2 and anti-Flag antibodies. (C) HEK293T cells were cotransfected with HA-p105 or Flag-ABIN2 (1 μg), along with increasing amounts of Flag-VP1- or HA-VP1-expressing plasmid (0, 1, 3, and 5 μg), and the empty Flag vector or empty HA vector (5, 4, 2, and 0 μg) was used in the transfection process. At 24 hpt, the expression levels of HA-p105 and Flag-ABIN2 proteins were analyzed by Western blotting with anti-HA and anti-Flag antibodies. (D and E) PK-15 cells were transfected with increasing amounts of Flag-VP1-expressing plasmid (0, 1, 3, or 5 μg) for 24 h. Empty Flag vector (5, 4, 2, or 0 μg) was used in the transfection process. (D) The expression levels of endogenous p105 and ABIN2 proteins were detected by Western blotting with anti-p105 and anti-ABIN2 antibodies. (E) The mRNA expression levels of endogenous TPL2, p105, and ABIN2 were measured by RT-qPCR. (F) PK-15 cells were transfected with FMDV L-expressing plasmid (3 μg), and the cells were collected at 0, 16, 32, and 48 hpt. The protein levels of eIF4G and Flag-L were determined by Western blotting. (G and H) PK-15 cells with or without infection with FMDV type A strain-WT, FMDV type O strain-WT, or FMDV type O strain-ΔL (MOI of 1) for 12 h. (G) The expression levels of endogenous TPL2 were detected by Western blotting. (H) The expression levels of TPL2 mRNA were measured by RT-qPCR.

FMDV decreases the expression of TPL2, but it was still unknown which viral protein plays a role in this process. Previous studies have shown that the FMDV structural proteins VP1 and VP2 and nonstructural protein L interact with TPL2. To identify viral proteins that downregulate TPL2 expression in these interacting proteins, Myc-tagged TPL2 and different concentrations of plasmids encoding Flag-tagged viral proteins were cotransfected into HEK293T cells. After 24 h, the abundance of Myc-TPL2 was detected by Western blotting. FMDV VP1 significantly decreased expression of Myc-TPL2 in a dose-dependent manner, while VP2 and L had no significant effect on TPL2 expression (Fig. 5A). PK-15 cells were then transfected with plasmids expressing different Flag-tagged viral proteins. The expression of endogenous TPL2 protein was further determined by Western blotting, and the same conclusions as those for exogenous were obtained (Fig. 5B). Subsequently, the effect of VP1 on the expression of two other proteins, p105 and ABIN2, in the trimer was evaluated, and all found that VP1 also downregulated the expression of exogenous and endogenous p105 and ABIN2 (Fig. 5C and D). To investigate whether the decrease in protein levels was the result of the specific decrease in mRNA expression, the levels of TPL2, p105, and ABIN2 mRNA in Flag-VP1-transfected cells were measured by RT-qPCR. There was no significant decrease in mRNA levels of the three proteins (Fig. 5E).

FMDV infection can cause cell protein synthesis to stop rapidly and limit cell protein synthesis. It is well known that L^pro^ can induce the cleavage of host eukaryotic translation initiation factor 4 (eIF4G), thereby inhibiting host protein synthesis (29, 30). To ensure the enzyme activity of L^pro^, PK-15 cells were transfected with Flag-L-expressing plasmids, and the kinetics of eIF4G protein levels were checked by Western blotting. L^pro^ cleaved eIF4G to produce cleavage bands (Fig. 5F), which was consistent with previous studies (31). To further confirm the impact of L^pro^ on the expression of TPL2, PK-15 cells were mock infected or infected with wild-type FMDV type A strain, wild-type FMDV type O strain, and FMDV-ΔL type O strain (leaderless virus), and TPL2 protein levels were measured and compared. The results showed that both FMDV type O and A strains inhibited the expression of TPL2 protein, while the expression of TPL2 protein was similar in the FMDV-WT- and FMDV-ΔL-infected cells (Fig. 5G). In addition, there was no significant difference in the transcription level of TPL2 between FMDV-WT- and FMDV-ΔL-infected cells (Fig. 5H). Thus, FMDV VP1 significantly inhibited the expression of TPL2, p105, and ABIN2 protein levels but had no effect on the transcription levels.

### FMDV VP1 protein specificity interacts with host TPL2.

**FIG 6:**
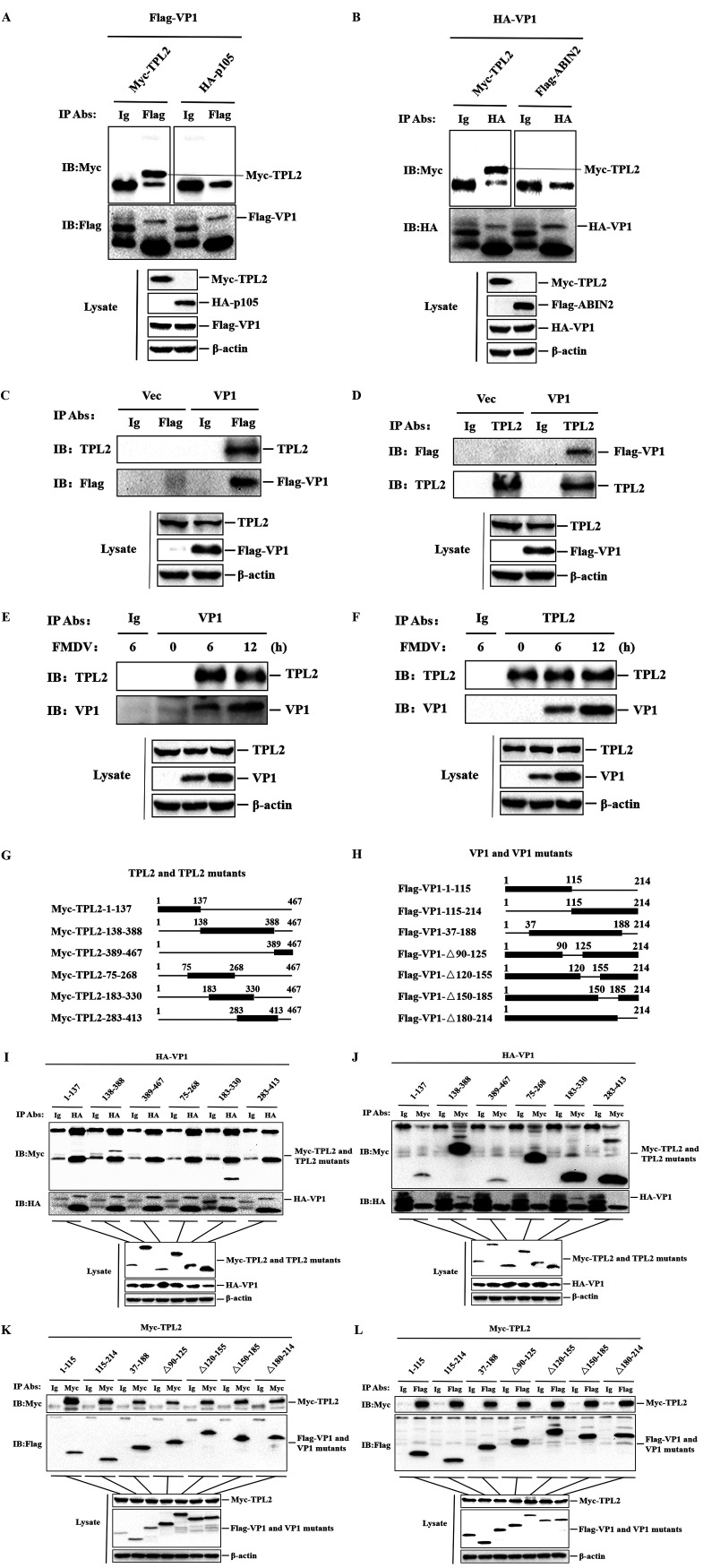
VP1 interacted with host TPL2 but not with p105 and ABIN2. (A) HEK293T cells were cotransfected with 8 μg Myc-TPL2- or HA-p105- and Flag-VP1-expressing plasmid. The cells were lysed at 24 hpt, and the lysates were immunoprecipitated with mouse IgG antibody or mouse anti-Flag antibody and subjected to Western blotting. Lysate and immunoprecipitation (IP) antibody-antigen complexes were analyzed by immunoblotting (IB) using anti-HA, anti-Myc, anti-Flag, and anti-β-actin antibodies. (B) The method is the same as that for panel A. The lysates were immunoprecipitated with mouse IgG antibody or mouse anti-HA antibody and subjected to Western blotting. (C and D) PK-15 cells were transfected with Flag-VP1-expressing plasmid (8 μg) or empty Flag vector (Vec; 8 μg) for 24 h. The cells were then lysed and immunoprecipitated with normal IgG antibody, anti-Flag antibody (C), or anti-TPL2 antibody (D). The lysate and IP antibody-antigen complexes were analyzed by IB using anti-Flag, anti-TPL2, or anti-β-actin antibodies. (E and F) PK-15 cells were mock infected or infected with FMDV (MOI of 1) for 6 or 12 h. MG132 (20 μM) was added to prevent the degradation of endogenous TPL2. The cells were then lysed and immunoprecipitated with normal IgG antibody, anti-VP1 antibody (E), or anti-TPL2 antibody (F). The lysate and IP antibody-antigen complexes were analyzed by IB using anti-TPL2, anti-VP1, or anti-β-actin antibodies. (G) Schematic representation showing a series of Myc-tagged truncated TPL2 mutants. (H) Schematic representation showing a series of Flag-tagged truncated VP1 mutants. (I and J) HEK293T cells were cotransfected with HA-VP1 (7 μg) and a series of Myc-tagged TPL2 truncated mutants expressing plasmids (7 μg). The cells were lysed at 24 hpt, and the lysates were immunoprecipitated with mouse IgG antibody, mouse anti-HA antibody (I), or mouse anti-Myc antibody (J) and subjected to Western blotting. (K and L) HEK293T cells were cotransfected with Myc-TPL2 (7 μg) and a series of Flag-tagged VP1 truncated mutants expressing plasmids (7 μg). The cells were lysed at 24 hpt, and the lysates were immunoprecipitated with mouse IgG antibody, mouse anti-Myc antibody (K), or mouse anti-Flag antibody (L) and subjected to Western blotting.

We next investigated the molecular mechanisms of immune escape mediated by FMDV structural protein VP1, focusing first on the interaction between VP1 and TPL2-p105-ABIN2. HEK293T cells were cotransfected with Flag-VP1- or hemagglutinin (HA)-VP1-expressing plasmids with Myc-TPL2-, HA-p105-, or Flag-ABIN2-expressing plasmids. Cells were lysed, and the lysates were immunoprecipitated with anti-Flag or anti-HA antibody and analyzed by Western blotting. As shown in Fig. 6A and B, Flag-VP1 and HA-VP1 pulled down Myc-TPL2, while HA-p105 and Flag-ABIN2 did not interact with VP1. To further investigate whether VP1 interacts with TPL2 under physiological conditions, PK-15 cells were transfected with Flag-VP1-expressing plasmid or empty Flag vector, and we conducted coimmunoprecipitation experiments with anti-Flag or anti-TPL2 antibody. We found that FDMV VP1 protein could interact with endogenous TPL2 (Fig. 6C and D). To confirm that the VP1-TPL2 interaction occurs in FMDV-infected cells, the coimmunoprecipitation assay was performed using the lysates from FMDV-infected cells. FMDV VP1 protein clearly pulled down TPL2 in the context of viral infection (Fig. 6E). The reverse immunoprecipitation assay was similarly performed using the anti-TPL2 antibody, and the results showed that TPL2 also immunoprecipitated VP1 (Fig. 6F). Collectively, these data firmly indicated that FMDV VP1 specificity interacted with TPL2 but not with p105 and ABIN2.

To further determine the binding region of the interaction between TPL2 and VP1, a series of truncation mutants of Myc-TPL2-expressing plasmids were generated through PCR-based site-directed mutagenesis (Fig. 6G). We conducted a detailed analysis for their interaction by constructing plasmids expressing Myc-tagged truncated mutants of TPL2 and plasmids expressing Flag-tagged truncated mutants of VP1 produced by our laboratory (Fig. 6H). HEK293T cells were cotransfected with HA-VP1-expressing plasmid and a series of plasmids expressing Myc-tagged truncated mutants of TPL2. Cell lysates were immunoprecipitated with anti-HA antibody and analyzed by Western blotting. As shown in Fig. 6I, TPL2 mutant with the 138–388 kinase region and TPL2 mutant with the 183–330 kinase region coprecipitated with HA-VP1, and the 183–330 kinase region of TPL2 contained a smaller region. Meanwhile, it was observed that the TPL2 mutant with the 75–268 kinase region and TPL2 mutant with the 283–413 kinase region failed to interact with VP1. Therefore, the region of their interaction was in the 268–283 kinase region of TPL2. A reverse immunoprecipitation experiment was subsequently performed using anti-Myc antibody, which obtained the same results (Fig. 6J). In addition, we cotransfected HEK293T cells with Myc-TPL2-expressing plasmid and a series of plasmids expressing Flag-tagged truncated mutants of VP1. Forward or reverse immunoprecipitation was subsequently performed using anti-Myc antibody or anti-Flag antibody and then analyzed by Western blotting. All plasmids expressing Flag-tagged truncated mutants of VP1 were coprecipitated with Myc-TPL2, suggesting that multiple regions are involved in the VP1-TPL2 interaction (Fig. 6K and L). All of these observations confirmed that VP1 interacted with host TPL2 but not with p105 and ABIN2, and the 268–283 kinase region of TPL2 was essential for this interaction.

### FMDV VP1 protein promotes K48-linked polyubiquitination of TPL2 and degrades TPL2 by the proteasome pathway.

**FIG 7:**
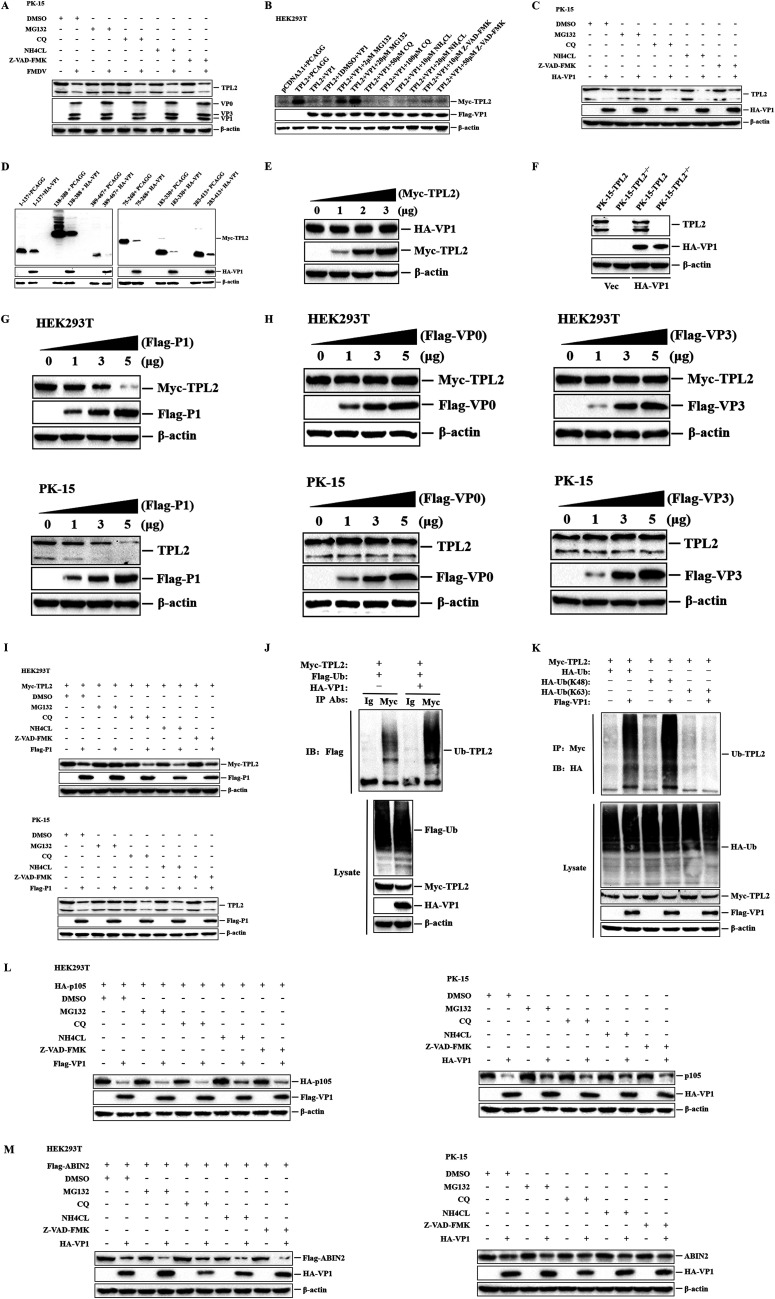
Degradation pathways of TPL2, ABIN2, and p105 induced by VP1 in HEK293T and PK-15 cells. (A) PK-15 cells treated with MG132 (20 μM), CQ (100 μM), NH_4_Cl (20 μM), or Z-VAD-FMK (50 μM) for 1 h and then with or without FMDV infection (MOI of 1) for 12 h. The expression levels of endogenous TPL2 and viral proteins were detected by Western blotting. DMSO, dimethyl sulfoxide. (B) HEK293T cells were cotransfected with Flag-VP1-expressing plasmid (2 μg) or empty Flag vector (2 μg) and Myc-TPL2-expressing plasmid (1 μg) and empty Myc vector (1 μg) and maintained in the presence or absence of MG132 (2 or 20 μM), CQ (50 or 100 μM), NH_4_Cl (10 or 20 μM), or Z-VAD-FMK (10 or 50 μM) for 36 h. The expression levels of Myc-TPL2 and Flag-VP1 proteins were detected by Western blotting. (C) PK-15 cells were transfected with HA-VP1-expressing plasmid (2 μg) or empty HA vector (2 μg) and maintained in the presence or absence of MG132 (20 μM), CQ (100 μM), NH_4_Cl (20 μM), or Z-VAD-FMK (50 μM) for 36 h. The expression levels of endogenous TPL2 and HA-VP1 proteins were detected by Western blotting. (D) HEK293T cells were cotransfected with HA-VP1 (5 μg) or empty HA vector (5 μg) and Myc-tagged TPL2 truncated mutant-expressing plasmids (2 μg). At 24 hpt, the cells were collected for Western blotting. (E) HEK293T cells were cotransfected with HA-VP1 (1 μg), along with increasing amounts of Myc-TPL2-expressing plasmid (0, 1, 2, and 3 μg), and the empty Myc vector (3, 2, 1, or 0 μg) was used in the transfection process. At 24 hpt, the cells were collected for Western blotting. (F) PK-15-TPL2 or PK-15-TPL2^−/−^ cells were transfected with HA-VP1-expressing plasmid (2 μg) or empty HA vector (2 μg). At 24 hpt, the cells were collected for Western blotting. (G and H) Similar transfection and immunoblotting analyses in HEK293T and PK-15 cells were performed as described for Fig. 5A and B. (I) HEK293T cells were cotransfected with Flag-P1 (3 μg) or empty Flag vector (3 μg) and Myc-TPL2-expressing plasmid (1 μg) and maintained in the presence or absence of MG132 (20 μM), CQ (100 μM), NH_4_Cl (20 μM), and Z-VAD-FMK (50 μM) for 36 h. The expression levels of Myc-TPL2 and Flag-P1 proteins were detected by Western blotting. PK-15 cells were transfected with Flag-P1-expressing plasmid (3 μg) or empty Flag vector (3 μg) and maintained in the presence or absence of MG132 (20 μM), CQ (100 μM), NH_4_Cl (20 μM), and Z-VAD-FMK (50 μM) for 36 h. The expression levels of endogenous TPL2 and Flag-P1 proteins were detected by Western blotting. (J) HEK293T cells were cotransfected with Flag-Ub-expressing (4 μg), Myc-TPL2-expressing (4 μg), HA-VP1-expressing (6 μg) plasmid, and empty HA vector (6 μg) for 20 h. The cells were then lysed and immunoprecipitated with mouse IgG antibody or mouse anti-Myc antibody and subjected to Western blotting. (K) HEK293T cells were cotransfected with HA-Ub, HA-K48-Ub, or HA-K63-Ub (4 μg), Myc-TPL2 (4 μg), Flag-VP1-expressing plasmid (6 μg), and empty Flag vector (6 μg) for 20 h. The cells were then lysed and immunoprecipitated with mouse anti-Myc antibody and subjected to Western blotting. (L) Degradation pathway of p105 induced by VP1 in HEK293T and PK-15 cells. The method is the same as that for panel I. (M) Degradation pathway of ABIN2 induced by VP1 in HEK293T and PK-15 cells. The method is the same as that for panel I.

To determine whether proteasomes, autophagosomes, lysosomes, or caspase-dependent pathways play roles in FMDV- or VP1-induced degradation of TPL2, the proteasome inhibitor MG132, the autophagy inhibitor chloroquine diphosphate (CQ), the lysosome inhibitor NH_4_CL, and the general caspase inhibitor benzyloxy carbonyl (Cbz)-l-Val-Ala-Asp (OMe)-fluoromethylketone (Z-VAD-FMK) were used to evaluate the inhibitive effects. PK-15 cells were treated with or without inhibitors and then mock infected or infected with FMDV. The expression of TPL2 at 12 hpi was detected by Western blotting. As shown in Fig. 7A, treatment of MG132 restored endogenous TPL2 levels in FMDV-infected cells, while CQ, NH_4_Cl, and Z-VAD-FMK had no such effect on TPL2 restoration (Fig. 7A). The effects of MG132, CQ, NH_4_Cl, or Z-VAD-FMK on VP1-induced reduction of TPL2 were also examined. Flag-VP1- and Myc-TPL2-expressing plasmids were cotransfected into HEK293T cells, and the cells were maintained in the presence or absence of the inhibitors. The expression of TPL2 was detected at 36 hpt by Western blotting. We found that VP1-mediated decrease of exogenous TPL2 protein was blocked by the proteasome inhibitor MG132, but the other three inhibitors (CQ, NH_4_Cl, and Z-VAD-FMK) had no effect on the degradation of exogenous TPL2 mediated by VP1 (Fig. 7B). Moreover, proteasome inhibitor MG132 also blocked VP1-induced endogenous TPL2 degradation (Fig. 7C), which provided more evidence that VP1 induced TPL2 degradation in a proteasomal-dependent manner. Next, to determine the functional domain of TPL2 degradation, various plasmids expressing truncated forms of TPL2 were cotransformed with HA-VP1-expressing plasmid or empty HA vector, and then the expression of various plasmids expressing truncated forms of TPL2 was detected at 24 h by Western blotting. Thus, we discovered that FMDV VP1 degraded the full functional domain of TPL2 (Fig. 7D). Since VP1 could inhibit TPL2, it attenuated the antiviral effect mediated by TPL2. We were interested in whether TPL2 could, in turn, inhibit the expression of VP1 and promote its antiviral effect. To test this hypothesis, the plasmids encoding Myc-TPL2 were used to confirm TPL2-induced reduction of VP1. For immunoblot analysis, Myc-TPL2 and HA-VP1 were cotransfected into HEK293T cells. As shown in Fig. 7E, TPL2 did not degrade VP1. Consistent with this, PK-15-TPL2 and PK-15-TPL2^−/−^ cells were transfected with HA-tagged VP1, and there is no significant difference in the expression level of VP1 in PK-15-TPL2 and PK-15-TPL2^−/−^ cells (Fig. 7F).

FMDV capsid precursor P1 is the precursor protein of VP1. P1 is finally processed into mature VP0, VP1, and VP3 proteins under the action of FMDV 3C protease (3C^pro^) (32, 33). To clarify whether the degradation of TPL2 is caused by the specificity of the VP1 protein in the P1 precursor, the effect of P1 on the expression level of TPL2 was first detected by Western blotting. It was observed that the abundance of exogenous and endogenous TPL2 proteins was reduced by the overexpression of P1 in a dose-dependent manner (Fig. 7G). However, FMDV VP0 and VP3 did not cause significant changes in the levels of exogenous and endogenous TPL2 proteins (Fig. 7H). The effects of MG132, CQ, NH_4_Cl, or Z-VAD-FMK on P1-induced degradation of TPL2 were also assessed. The results showed that incubation of MG132 restored exogenous and endogenous TPL2 levels in P1-overexpressed cells, while CQ, NH_4_Cl, and Z-VAD-FMK had no such effect on TPL2 restoration (Fig. 7I). These results indicated that the degradation of TPL2 induced by P1 was caused by the specificity of the VP1 protein.

Furthermore, we subsequently checked the effect of VP1 on the ubiquitination of TPL2, which serves as the essential sign of protein degradation mediated by the ubiquitin-proteasome system. We cotransfected a Myc-TPL2-expressing plasmid with a Flag-Ub-expressing plasmid, along with HA-VP1-expressing plasmid or empty HA vector simultaneously. Immunoprecipitation experiments were conducted with anti-Myc antibody, and the ubiquitination levels of TPL2 were determined by Western blotting. Overexpression of VP1 enhanced the ubiquitination of TPL2, which corresponded to the decrease of TPL2 protein expression (Fig. 7J). Similarly, immunoprecipitation analysis of ubiquitin also demonstrated that overexpression of VP1 significantly enhanced K48- rather than K63-linked polyubiquitination of TPL2 (Fig. 7K). Together, these results indicated that FMDV VP1 specifically enhanced K48-linked polyubiquitination of TPL2 and degraded TPL2 by the proteasome pathway.

The above-described results showed that FMDV VP1 can also inhibit the expression of p105 and ABIN2. We sought to determine the pathways by which VP1 degrades p105 and ABIN2. Treatment with proteasome inhibitor MG132, autophagy inhibitor CQ, the lysosome inhibitor NH_4_CL, and caspase inhibitor Z-VAD-FMK did not significantly block the exogenous and endogenous protein reduction (Fig. 7L and M). These results suggested that VP1-induced reduction of p105 and ABIN2 was independent of proteasome, autophagy, lysosome, and caspase pathways.

### FMDV VP1 protein destroys the stability of the TPL2-p105-ABIN2 trimer complex.

**FIG 8:**
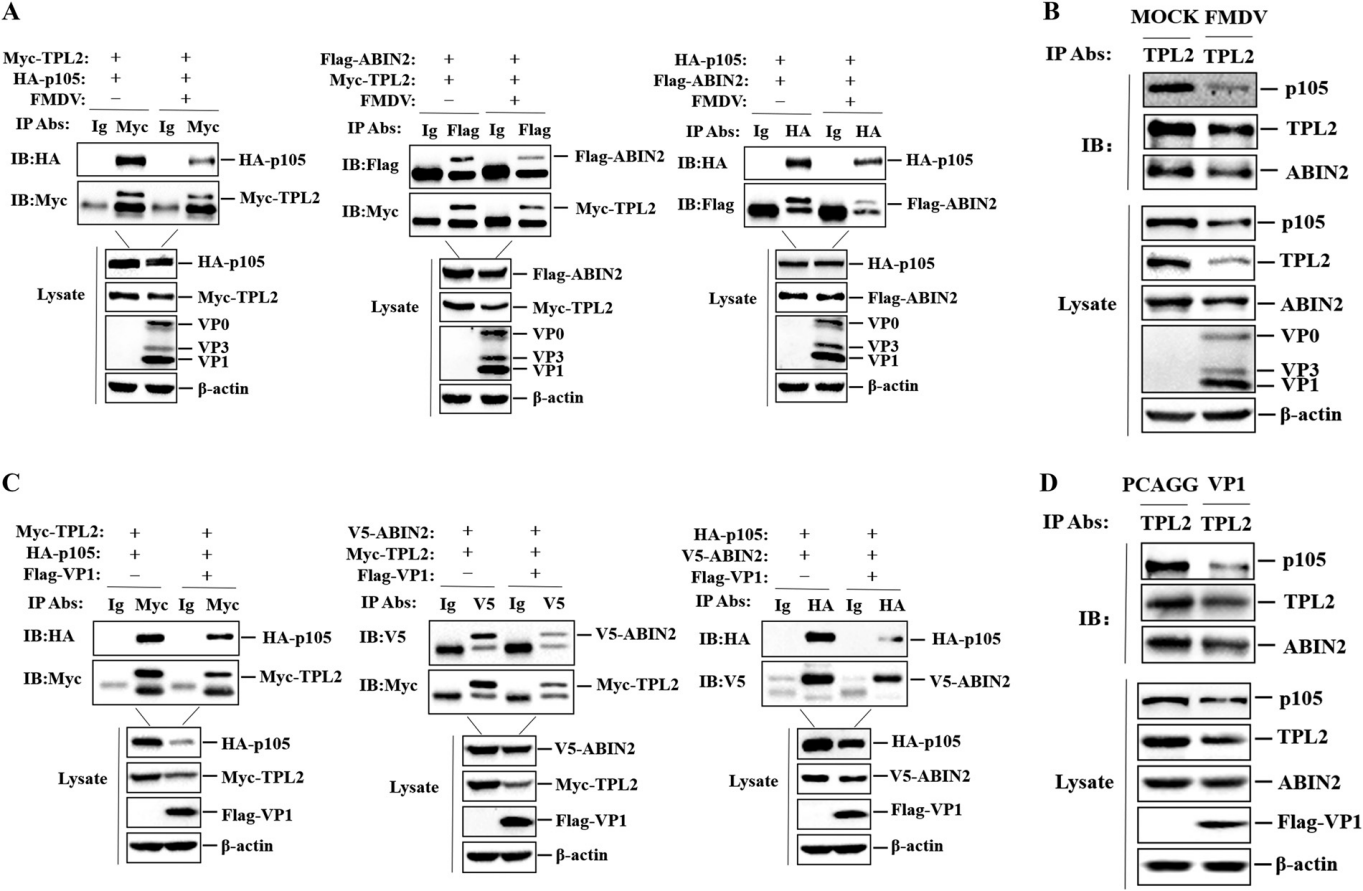
FMDV and FMDV VP1 destroy the stability of the TPL2-p105-ABIN2 trimer complex. (A) PK-15 cells were cotransfected in pairs with plasmid expressing Myc-TPL2 (5 μg), HA-p105 (5 μg), or Flag-ABIN (5 μg) for 24 h, with or without FMDV infection (MOI of 1) for 12 h. The cells were lysed, and the lysates were immunoprecipitated with anti-Myc antibody, mouse anti-Flag antibody, anti-HA antibody, and IgG antibody and subjected to Western blotting. (B) PK-15 cells with or without FMDV infection (MOI of 1) for 12 h. The cells were lysed and immunoprecipitated with mouse anti-TPL2 antibody. The lysate and IP antibody-antigen complexes were analyzed by IB using anti-TPL2, anti-p105, anti-ABIN2, anti-FMDV, and anti-β-actin antibodies to measure the expression of endogenous TPL2, p105, and ABIN2 separately. (C) HEK293T cells were cotransfected in pairs with plasmid expressing Myc-TPL2 (5 μg), HA-p105 (5 μg), and V5-ABIN2 (5 μg). Flag-VP1-expressing plasmid (8 μg) and empty Flag vector (8 μg) were transfected at the same time. At 24 hpt, the cells were lysed and the lysates were immunoprecipitated with anti-Myc antibody, anti-V5 antibody, anti-HA antibody, and mouse normal IgG antibody. (D) PK-15 cells were transfected with Flag-VP1-expressing plasmid (8 μg) and empty Flag vector (8 μg) for 24 h. The cells were lysed and immunoprecipitated with anti-TPL2 antibody and subjected to Western blotting. The lysate and IP antibody-antigen complexes were analyzed by IB to measure the expression of endogenous TPL2, p105, and ABIN2 separately.

A previous study showed that TPL2 forms a complex with p105 and ABIN2 in stable states, and TPL2 is inactive in this state (34). TPL2 is active only when released from the trimer complex, thereby triggering downstream signal transductions. Therefore, it is necessary to evaluate the effect of FMDV and FMDV VP1 protein on the stability of the trimer. PK-15 cells were cotransfected in pairs with Myc-TPL2-, HA-p105-, and Flag-ABIN2-expressing plasmids. After 24 h, the cells were infected with FMDV for 12 h. FMDV reduced the interactions of TPL2-p105, TPL2-ABIN2, and p105-ABIN2 (Fig. 8A). To further determine the effect of FMDV on the stability of the trimer, PK-15 cells were infected with FMDV, and the complex was immunoprecipitated with anti-TPL2 antibody and analyzed by Western blotting. As with the experiments using exogenous protein expression, the interaction between endogenous TPL2, p105, and ABIN2 was also reduced in the context of virus replication (Fig. 8B).

Because VP1 is a viral protein that plays a role in the reduction of TPL2 expression induced by FMDV, we speculated that VP1 could also reduce the stability of the trimer complex. To test this hypothesis, plasmids encoding Myc-tagged TPL2, HA-tagged p105, and V5-tagged ABIN2 were cotransfected in pairs into HEK293T cells, plasmids encoding Flag-tagged VP1 were transfected together, and immunoprecipitation was performed with specific antibodies. As shown in Fig. 8C, VP1 inhibited the pairwise binding of TPL2-p105, TPL2-ABIN2, and p105-ABIN2. Subsequently, PK-15 cells were transfected with Flag-VP1-expressing plasmid or empty Flag vector. The lysates were immunoprecipitated with anti-TPL2 antibody and analyzed by Western blotting. It was observed that VP1 reduced the endogenous interaction of TPL2, p105, and ABIN2 proteins (Fig. 8D). Taken together, these results indicated that FMDV VP1 protein disrupted the interaction of TPL2, p105, and ABIN2 by targeting TPL2 and destroying the stability of the trimer complex.

## DISCUSSION

TPL2 is a member of the mitogen-activated protein 3 kinase (MAP3K) family and plays an important role in the host defense against pathogen infection (35). Past research on TPL2 has focused on fighting bacterial infections. For example, TPL2 promotes Helicobacter pylori-induced extracellular signal-regulated kinase (ERK) activation and IL-8 expression, which prevents bacterial infection (36); TPL2 promotes innate cell recruitment and effector T-cell differentiation to limit Citrobacter rodentium burden and dissemination (37); and TPL2 can promote the production of IL-1β, which is essential for host defense against Listeria monocytogenes (38). However, the role of TPL2 in defending against viral pathogens is still poorly understood. A study in recent years has shown that TPL2 resisted the invasion of influenza viruses by promoting the production of IFN-λ and interferon-stimulated genes and inducing antigen-specific CD8^+^ T-cell responses (39). This suggests that TPL2 also plays a broader role in antiviral innate immunity.

Gain-of-function and loss-of-function experiments confirmed the antiviral role of TPL2 during FMDV infection *in vitro*. However, TPL2 gene knockout in PK-15 cells significantly promoted the replication of FMDV. We also found that TPL2 has a positive regulatory effect on FMDV-induced interferons and other antiviral factors. Subsequently, we constructed TPL2 knockout mice for *in vivo* experiments to further verify the experimental results *in vitro* and obtained the same conclusions. In this study, we elucidated the antiviral activity of TPL2 on FMDV for the first time *in vitro* and *in vivo*. TPL2 exerted its antiviral effect by promoting the production of interferons and other antiviral cytokines induced by FMDV and ultimately inhibited FMDV replication.

During the process of evolution, FMDV has acquired various strategies to antagonize host innate immune response, forming a unique immune escape mechanism that enables it to survive host antiviral defenses (1). However, the mechanisms involved are not fully understood. One of the potential strategies used by FMDV to antagonize host innate immune responses involves interaction between proteins encoded by the virus and host proteins to disrupt the function of the host proteins and reduce the production of host protein-mediated antiviral cytokines that inhibit viral replication. FMDV VP3 interacts with Janus kinase 1 (JAK1) and JAK2, destroys the assembly of the JAK1 complex, and degrades JAK1 through the lysosomal pathway, thereby inhibiting the type II IFN signaling pathway (40); FMDV 2B interacts with RIG-I and reduces its expression, effectively antagonizing the RIG-I-induced antiviral effect (41); and FMDV 3A interacts with RIG-1, melanoma differentiation-associated protein 5 (MDA5), and virus-induced signaling adaptor (VISA), inhibiting their expression on a transcriptional level and thereby inhibiting the induction of the antiviral cytokine IFN-β (42). Notably, we demonstrated that FMDV infection significantly inhibited TPL2 expression *in vivo* and *in vitro* and also inhibited the expression of p105 and ABIN2. However, before the present study, it was unknown which viral protein played a role in this process.

In recent years, it has been discovered that the FMDV structural protein VP1 also plays an important role in regulating the host immune response (43). For example, the heat shock protein DNAJA3 interacts with VP1, reducing its antagonistic effect on the IFN-β signaling pathway by inducing lysosomal degradation of VP1, thereby inhibiting FMDV virus replication (44). VP1 interacts with host ribosomal protein SA (RPSA) to maintain activation of the MAPK signaling pathway and promote virus replication (45); VP1 protein inhibits type I interferon production by interacting with host cell protein Sorcin (46). Here, we describe that among the three viral proteins L, VP1, and VP2 that interact with TPL2, only VP1 specifically reduced the protein level of TPL2 in a dose-dependent manner while also reducing the protein levels of p105 and ABIN2, although it has no effect on the mRNA levels of the three proteins. FMDV L^pro^ is well known as a viral proteinase. L^pro^ can induce the cleavage of host eukaryotic translation initiation factor 4γ (eIF4G), limiting the synthesis of host proteins (47). Interestingly, we found that FMDV L^pro^ did not induce the reduction of TPL2. Subsequently, VP1 was observed to interact with TPL2 but not with p105 and ABIN2. This finding was further confirmed in cells infected with FMDV. This implies that the VP1 protein specifically interacted with TPL2 and then induced the destruction of TPL2. The 268- to 283-amino-acid region in the TPL2 kinase domain was essential for its interaction with VP1.

VP1 regulates the stability of TPL2, p105, and ABIN2. Protein stability is regulated by ubiquitin-proteasome-mediated degradation, autophagy/lysosome-mediated degradation, or caspase-mediated degradation (48–50). In this study, we discovered that VP1-mediated degradation of TPL2 was blocked by the proteasome inhibitor MG132 but not by the autophagy inhibitor CQ, the lysosomal inhibitor NH_4_CL, or the caspase inhibitor Z-VAD-FMK. In addition, VP1 could degrade the entire segment of TPL2, but TPL2 had no obvious effect on the expression of VP1. Many viral proteins can modify host proteins through ubiquitination, leading to the degradation of host proteins through the proteasome pathway (51–53). In the present study, we demonstrated that VP1 indeed significantly promoted the ubiquitination of TPL2, which corresponded to the decrease of TPL2 protein levels. Further study indicated that VP1 promoted K48- rather than K63-linked polyubiquitination of TPL2. In addition, although VP1 significantly induced the reduction of p105 and ABIN2 in cultured cells, VP1-induced degradation of p105 and ABIN2 occurred in a proteasome-, lysosome-, and caspase-independent manner. It indicates that VP1 represses p105 and ABIN2 expression by some complicated mechanisms.

The stability of the TPL2-p105-ABIN2 trimer complex is critical to the activation of TPL2. TPL2 is active only when released from the trimer complex, and it triggers downstream signal transduction to function (54). Therefore, it is necessary to evaluate the influence of FMDV and FMDV VP1 protein on the stability of trimer. In the present study, we observed that FMDV infection disrupted the interaction of the TPL2, p105, and ABIN2 proteins, destroying the stability of the trimer complex. Further research revealed that FMDV VP1 also destroyed the stability of the trimer complex.

**FIG 9:**
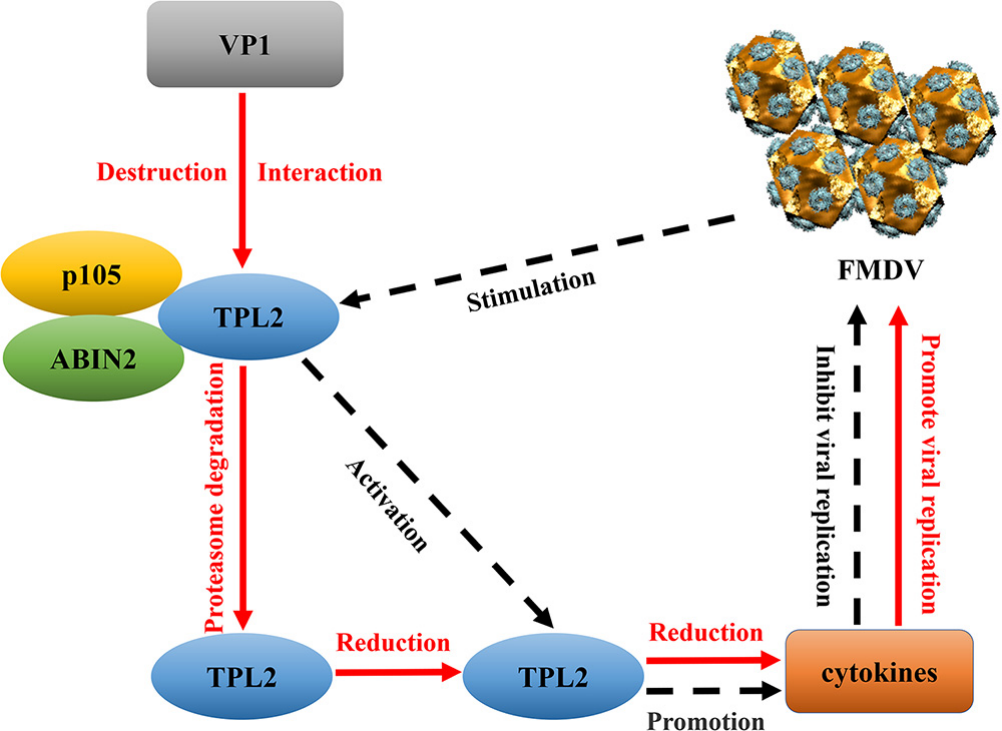
Schematic representation showing the model of TPL2 involvement in FMDV replication. TPL2 inhibits FMDV replication by promoting FMDV-induced interferons and other antiviral factors. FMDV VP1 protein destroys the stability of TPL2 trimer by targeting TPL2 degradation, leading to the reduction of TPL2, which can induce the production of antiviral cytokines, thereby facilitating FMDV replication.

In conclusion, the present study determined the antiviral effect of TPL2 during FMDV infection. TPL2 inhibited FMDV replication by promoting FMDV-induced interferons and other antiviral factors. We further identified a novel immune escape mechanism for FMDV (Fig. 9). Our data also demonstrated that the FMDV VP1 protein promoted K48-linked polyubiquitination of TPL2 and degraded TPL2 through the proteasome pathway, while VP1 specifically interacted with TPL2 and destroyed the stability of the TPL2-p105-ABIN2 trimer. These findings suggest that the FMDV VP1 protein destroyed the stability of TPL2 trimer by targeting TPL2 degradation, thereby decreasing the amount of TPL2 that can be released from the trimer and activated to induce the downstream production of antiviral cytokines, enhancing the replication of FMDV. This provided us with new insights into the pathogenesis of FMDV.

## MATERIALS AND METHODS

### Cells, viruses, and viral infection.

Porcine kidney PK-15 cells and human embryonic kidney 293T (HEK293T) cells were cultured in Dulbecco’s modified Eagle medium (DMEM; Gibco) supplemented with 10% heat-inactivated fetal bovine serum (FBS; Gibco), 1% penicillin-streptomycin and maintained at 37°C with 5% CO_2_. PK-15 cells are susceptible to FMDV infection and were used for viral infection experiments in the present study. HEK293T cells are easily transfected and were used to investigate exogenous protein-protein interactions as well as to express viral and host proteins. FMDV type A strain A/GDMM/CHA/2013 was used for all viral challenge experiments. FMDV type A strain A/GDMM/CHA/2013 and type O strain O/BY/CHA/2010 were obtained from the National Foot and Mouth Diseases Reference Laboratory, Lanzhou Veterinary Research Institute, Chinese Academy of Agricultural Sciences. FMDV type O strain-WT was generated from the full-length serotype O/BY/CHA/2010 infectious clones, and FMDV type O strain-ΔL (leaderless virus) was generated from the infectious clones lacking the Lb coding region as described previously (29). For the virus infection experiment, PK-15 cells were washed with serum-free medium and incubated with strain A/GDMM/CHA/2013 of a specified MOI at 37°C for 1 h for adsorption. After adsorption, the inoculum was removed and replaced with a virus maintenance medium containing 2% FBS to continue the cultures. The infected cells were collected and washed twice with phosphate-buffered saline (PBS) for subsequent experiments.

### Reagents and antibodies.

MG132, CQ, NH_4_Cl, and Z-VAD-FMK were purchased from Sigma-Aldrich (St. Louis, MO, USA). Actinomycin D was purchased from MCE. The commercial antibodies used in this study were mouse anti-Flag monoclonal antibody, mouse anti-Myc monoclonal antibody, mouse anti-HA monoclonal antibody (all from Sigma-Aldrich), mouse anti-V5 monoclonal antibody (Proteintech), mouse anti-TPL2 monoclonal antibody (Santa Cruz Biotechnology), mouse anti-eIF4G monoclonal antibody (Santa Cruz Biotechnology), rabbit anti-p105 polyclonal antibody (Cell Signaling Technology), rabbit anti-ABIN2 polyclonal antibody (Proteintech), anti-β-actin monoclonal antibody (Thermo Fisher Scientific), goat anti-mouse IgG antibody (Proteintech), and goat anti-rabbit IgG antibody (Proteintech). Rabbit anti-FMDV polyclonal antibody was prepared in our laboratory, and Western blotting showed three protein bands of VP0 (40 kDa), VP1 (25 kDa), and VP3 (26 kDa). Mouse anti-VP1 monoclonal antibody was provided by the OIE FMD reference laboratory of China (Lanzhou, Gansu, People’s Republic of China).

### Plasmid constructs and transfection.

Full-length cDNA of TPL2 was amplified from PK-15 cells and cloned into the pcDNA3.1/myc-His(−)A vector (Invitrogen, Carlsbad, CA, USA) to generate plasmids expressing Myc-tagged TPL2 (Myc-TPL2). A series of Flag-tagged VP1 truncated mutants expressing plasmids were generated by site-directed mutagenesis PCR in our laboratory as described previously (55). FMDV full-length viral cDNA was inserted into the p3×Flag-CMV-7.1 vector (Sigma-Aldrich) to construct plasmids expressing Flag-tagged viral proteins as described previously (56). The Flag-Ub, Myc-TPL2, HA-p105, Flag-ABIN2, and V5-ABIN2 plasmids, as well as various HA-tagged ubiquitinated plasmids used in this study, all were made by our laboratory. All constructed plasmids were sequenced and analyzed to ensure that the target gene was inserted into the vector plasmid accurately. All transfection experiments were performed with Lipofectamine 2000 (Invitrogen) as the transfection agent, with the specific transfection method performed according to the manufacturer’s instructions.

### Establishment of a TPL2 knockout PK-15 cell line using the CRISPR/Cas9 system.

Using gene ID 100622217 of the TPL2 gene in the NCBI database, two single-guide RNAs (sgRNA) were designed by selecting the sequence with higher score as the target site in the Ensembl genome database (http://asia.ensembl.org/Sus_scrofa/Transcript/Summary?db=core;g=ENSSSCG00000020705;r=10:40709927-40746355;t=ENSSSCT00000033089). To construct the guide RNA expression plasmid, complementary oligonucleotides encoding gRNA1 (5′-TTCCATAATGTCTATTACATCGG-3′) and gRNA2 (5′-AGATCCCAGATTCCTGGGGTCGG-3′) were annealed and cloned into BsmBI sites in lentiGuide-EGFP. Lentivirus particles were produced by cotransfection of recombinant plasmids and lentivirus packaging auxiliary plasmids. The supernatant of the packaged virus was collected and purified by 4°C ultracentrifugation. PK-15 cells in logarithmic phase were seeded into six-well plates with a fusion degree of 5 × 10^5^ per well and then transduced with lentivirus. Polybrene was added to a final concentration of 6 μg/ml. At 12 hpi, cell supernatants were changed to fresh medium, and culture was continued for 72 h. The clones with fluorescence were separated by flow cytometry. The genomic DNA of the cells cultured from a single-cell clone was analyzed by PCR using the pig TPL2 check primers (forward, 5′-GGACAGCAGGTGAAACGCATCT-3′; reverse, 5′-CCACAGCCATAGCCACAACC-3′). Frame-shifting mutations of alleles in established monoclonal cell lines were identified by sequencing analysis. Western blotting confirmed that TPL2 was completely knocked out in the established cell line. PK-15 cells transfected with lentiGuide-EGFP empty vector were used as a control.

### Establishment of TPL2 knockout mice using the CRISPR/Cas9 system.

TPL2^−/−^ and TPL2^+/+^ mice on the C57BL/6J background were generated at the Shanghai Model Organisms Center, Inc. (Shanghai, China), using CRISPR/Cas9 technology to repair nonhomologous recombination to introduce mutations resulted in frame-shifting of the TPL2 gene protein and loss of function. PCR products were sequenced to analyze the frame-shifting of the target gene protein in F0 mice. Positive F0 generation mice were selected to mate with wild-type C57BL/6J mice to obtain F1 generation heterozygous mice. Genotyping by PCR was performed using a combination of the following primers: 1, 5′-GCCTGGGCCCACCTATCTCTTCTC-3′; 2, 5′-ACCAGTTTGCACGCCATTCTTTTC-3′; 3, 5′-ACACCAAGCCCATCCCATAGTAGG-3′; and 4, 5′-GAGGCAGCACATCCAACAAACACG-3′. Amplification of the wild-type allele with primers 1 and 2 gave rise to a 740-bp fragment, while amplification of the mutant allele primers 3 and 4 led to a 437-bp fragment. Western blotting confirmed that TPL2 was completely knocked out in the established mice. TPL2^–/–^ mice were bred under specific-pathogen-free conditions. Three-day-old male and female mice were randomly allocated for each experimental group, and littermates were used as controls.

### Immunoblot analysis.

For Western blotting, cells were lysed in lysis buffer (Beyotime Biotechnology). Cell lysates were then denatured by boiling in a 100°C metal bath and centrifuged at 12,000 rpm for 10 min to remove cell fragments. Target proteins were resolved by SDS-PAGE and transferred onto an Immobilon-P membrane (Millipore). This was then incubated with 5% skim milk at room temperature for 2 h and incubated with appropriate primary antibodies and secondary antibodies as described previously (57). The antibody-antigen complexes were visualized using enhanced chemiluminescence (Thermo Fisher Scientific, Waltham, MA, USA).

### RNA interference.

Small interfering RNA (siRNA) was synthesized by GenePharma (Shanghai, China) and used for the knockout of endogenous TPL2 in cells. PK-15 cells were cultured in six-well plates to a confluence of 80% to 90% and then transfected with 100 pM TPL2 siRNA or an equivalent amount of NC siRNA with Lipofectamine 2000 reagent. The follow-up experiment was carried out at 48 h posttransfection.

### Coimmunoprecipitation assay.

HEK293T or PK-15 cells were seeded in a 10-cm dish. The monolayer of cells was transfected with different doses of plasmid, infected with FMDV, or mock infected. The cells were lysed with 1 ml lysis buffer (50 mM Tris [pH 8.0], 150 mM NaCl, 5 mM EDTA, 50 mM EDTA free EASY pack [27423500; Roche], 0.5% Nonidet P-40), and then 1 to 2 μl of proper antibodies or 0.4 μl of control IgG were used to immunoprecipitate the interacted proteins by 50% (vol/vol) slurry of GammaBind G Plus-Sepharose (GE Health Care Life Sciences, Piscataway, NJ, USA) overnight at 4°C. The precipitates were analyzed by immunoblot assay as described above.

### RNA extraction and RT-qPCR.

Total RNA was extracted using TRIzol reagent (Invitrogen). Isolated RNA was reverse transcribed into cDNA using M-MLV reverse transcriptase (Promega) and random hexamer primers (TaKaRa). The generated cDNA was used as the template for expression analysis of all target genes by qPCR. The Mx3005P qPCR system (Agilent Technologies) and SYBR Premix *Ex Taq* reagents (TaKaRa) were used in RT-qPCR experiments to quantify the expression of various mRNAs. The glyceraldehyde-3-phosphate dehydrogenase (GAPDH) gene was used as the internal control. The relative expression of mRNA was calculated based on the comparative cycle threshold (2^−ΔΔ^*^CT^*) method (58).

RT-qPCR was used to quantify the viral genome copy numbers in PK-15 cells infected with FMDV as previously described (59). All of the experiments were repeated in triplicate, with similar results. Data represent results from one representative triplicate experiment.

### Pharmacological inhibition of proteasome, lysosome, and caspase inhibitor pathway by their specific inhibitors.

PK-15 cells were cultured in 12-well plates to a confluence of 70% to 80% and then treated with proteasome inhibitor MG132 (20 μM), autophagy inhibitor chloroquine diphosphate (CQ; 100 μM), lysosome inhibitor NH_4_Cl (20 μM), or caspase inhibitor benzyloxycarbony (Cbz)-l-Val-Ala-Asp (OMe)-fluoromethylketone Z-VAD-FMK (50 μM) for 1 h. After the treatment, the cells were mock infected or infected with FMDV (MOI of 1) for 12 h or PK-15 cells were cultured in 12-well plates to a confluence of 80% to 90% and then transfected with HA-VP1 (Flag-VP1)- or Flag-P1-expressing plasmid or empty HA (Flag) vector using Lipofectamine 2000 and maintained in the presence or absence of MG132 (20 μM), CQ (100 μM), NH_4_Cl (20 μM), or Z-VAD-FMK (50 μM) for 36 h. As for HEK293T cells, the cells were cultured in 12-well plates to a confluence of 80% to 90% and then cotransfected with Myc-TPL2-expressing plasmid or empty Myc vector and Flag-VP1-expressing plasmid or empty Flag vector using Lipofectamine 2000 and maintained in the presence or absence of MG132 (2 or 20 μM), CQ (50 or 100 μM), NH_4_Cl (10 or 20 μM), or Z-VAD-FMK (10 or 50 μM) for 36 h, or HEK293T cells were cultured in 12-well plates to a confluence of 80% to 90%, cotransfected with HA-p105- or Flag-ABIN2-expressing plasmid and Flag-VP1 (HA-VP1)- and Flag-P1-expressing plasmid or empty Flag (HA) vector using Lipofectamine 2000, and maintained in the presence or absence of MG132 (20 μM), CQ (100 μM), NH_4_Cl (20 μM), or Z-VAD-FMK (50 μM) for 36 h. The collected cells were then subjected to Western blotting.

### Statistical analysis.

The significance of the results between the experiments was analyzed using Prism 6.01 software (GraphPad, San Diego, CA, USA). All data are presented as mean values ± standard errors (SEs) from three independent experiments. A *P *value of *<*0.05 (*) was considered statistically significant, and a *P *value of *<*0.01 (**) was considered highly statistically significant.
